# Cardiac abnormalities in a young athlete with severe pectus excavatum: a case report of a diagnostic loop between anatomical distortion and cardiomyopathy

**DOI:** 10.1093/ehjcr/ytag364

**Published:** 2026-05-21

**Authors:** Massimo Mapelli, Filippo Maria Rubbo, Gian Luca Ragazzoni, Sara Moscatelli, Paolo Certini, Edoardo Conte, Gianluca Pontone, Piergiuseppe Agostoni

**Affiliations:** Centro Cardiologico Monzino, IRCCS, Via Carlo Parea 4, 20138 Milan, Italy; Centro Cardiologico Monzino, IRCCS, Via Carlo Parea 4, 20138 Milan, Italy; Center for Diagnosis and Treatment of Cardiomyopathies, Cardiovascular Department, Azienda Sanitaria Universitaria Giuliano-Isontina (ASUGI), University of Trieste, Via Costantino Costantinides 2, 34128 Trieste, Italy; Department of Medical Biotechnologies, Sports Cardiology and Rehab Unit, University of Siena, Viale Mario Bracci 16, 53100 Siena, Italy; Centre for Inherited Cardiovascular Disease, Great Ormond Street Hospital, Great Ormond Street, London WC1N 3JH, UK; Institute of Cardiovascular Sciences, University College London, Gower Street, London WC1E 6BT, UK; Centro Cardiologico Monzino, IRCCS, Via Carlo Parea 4, 20138 Milan, Italy; Department of Clinical Cardiology and Cardiovascular Imaging, IRCCS Galeazzi-Sant'Ambrogio Hospital, Via Cristina Belgioioso 173, 20157 Milan, Italy; Centro Cardiologico Monzino, IRCCS, Via Carlo Parea 4, 20138 Milan, Italy; Department of Biomedical, Surgical and Dental Sciences, University of Milan, Via Festa del Perdono 7, 20122 Milan, Italy; Centro Cardiologico Monzino, IRCCS, Via Carlo Parea 4, 20138 Milan, Italy; Department of Clinical Sciences and Community Health, Cardiovascular Section, University of Milan, Via Festa del Perdono 7, 20122 Milan, Italy

**Keywords:** Pectus excavatum, Cardiac magnetic resonance, Non-dilated left ventricular cardiomyopathy, NDLVC, Athlete, Sports cardiology, Ventricular ectopies, Case report

## Abstract

**Background:**

Pectus excavatum (PEX) is the most common congenital chest wall deformity, occasionally associated with cardiac displacement and mild functional impairment. Its role in masking or mimicking cardiomyopathy remains poorly defined, particularly in athletes.

**Case summary:**

We report the case of a 24-year-old asymptomatic male athlete with severe PEX, frequent monomorphic premature ventricular contractions (PVCs), and mildly reduced left ventricular ejection fraction (LVEF). Despite successful PVC ablation and absence of genetic mutations, fibrosis, or structural abnormalities on cardiac magnetic resonance and electroanatomic mapping, the patient maintained borderline LV dysfunction. These findings raised the possibility of either a mild non-dilated left ventricular cardiomyopathy (NDLVC) or a reversible functional impairment due to chest wall distortion. Based on Italian guidelines, the patient was deemed ineligible for competitive sports. However, we explored how this case might be handled in other European countries with more permissive or flexible criteria.

**Discussion:**

This case underscores the diagnostic uncertainty posed by overlapping anatomical and functional findings, and the potentially profound impact of disqualification from sport on young athletes. It also reveals discrepancies in national approaches to eligibility and the interpretation of borderline findings. The case prompts reflection on the need for more harmonized guidelines and, in selected cases, structured shared decision-making processes involving expert centres and the athlete.

**Conclusion:**

In the evolving context of NDLVC, this case highlights the difficulty of acting in rigid frameworks and diagnostic loops, advocating instead for individualized, responsible decision-making that balances safety with athlete well-being.

Learning points

- Severe pectus excavatum may mimic mild non-dilated left ventricular (LV) cardiomyopathy, leading to diagnostic uncertainty, especially in athletes
- In this case, despite successful ablation and absence of fibrosis or genetic mutations, mild LV dysfunction persisted, suggesting functional impairment from anatomical distortion
- Differences among national eligibility criteria highlight the need for harmonized, shared decision-making models in borderline cases of athletes with structural chest deformities

## Introduction

Pectus excavatum (PEX), commonly known as funnel chest, is the most frequent anterior chest wall deformity in children, occurring in approximately 1 in every 400 Caucasian male births. The condition is notably more common in males, with a male-to-female ratio of 4:1.^1^ The deformity is likely due to disproportionate overgrowth in the costochondral regions, which results in a concave shape of the anterior chest wall and can displace the heart leftward due to sternal pressure. Aside from mitral valve prolapse, in these subjects, structural abnormalities within the heart are generally considered rare. Nonetheless, PEX is associated with symptoms such as fatigue, rapid breathing, chest discomfort, and dyspnoea.^1^ Recent trials, involving also cardiac magnetic resonance (CMR) evaluation,^2^ showed how patients with PEX deformity have varying levels of distortion of the right ventricle (RV), associated with a 6% reduction in resting RV ejection fraction (RVEF) relative to controls. The degree of physiological impairment is likely related to the degree of geometrical distortion of the RV due to the sternal compression.

We report on a 24-year-old male athlete presenting with frequent monomorphic ventricular contractions (PVCs), initially discovered during a preparticipation screening program (PPS). The PPS is mandatory for sports eligibility and is repeated at least annually. It includes medical history, physical examination, resting electrocardiogram, and stress exercise testing; additional tests are reserved for athletes with abnormal findings.^3^

The case highlights diagnostic findings, therapeutic interventions, and follow-up data.

## Summary figure

**Figure:**
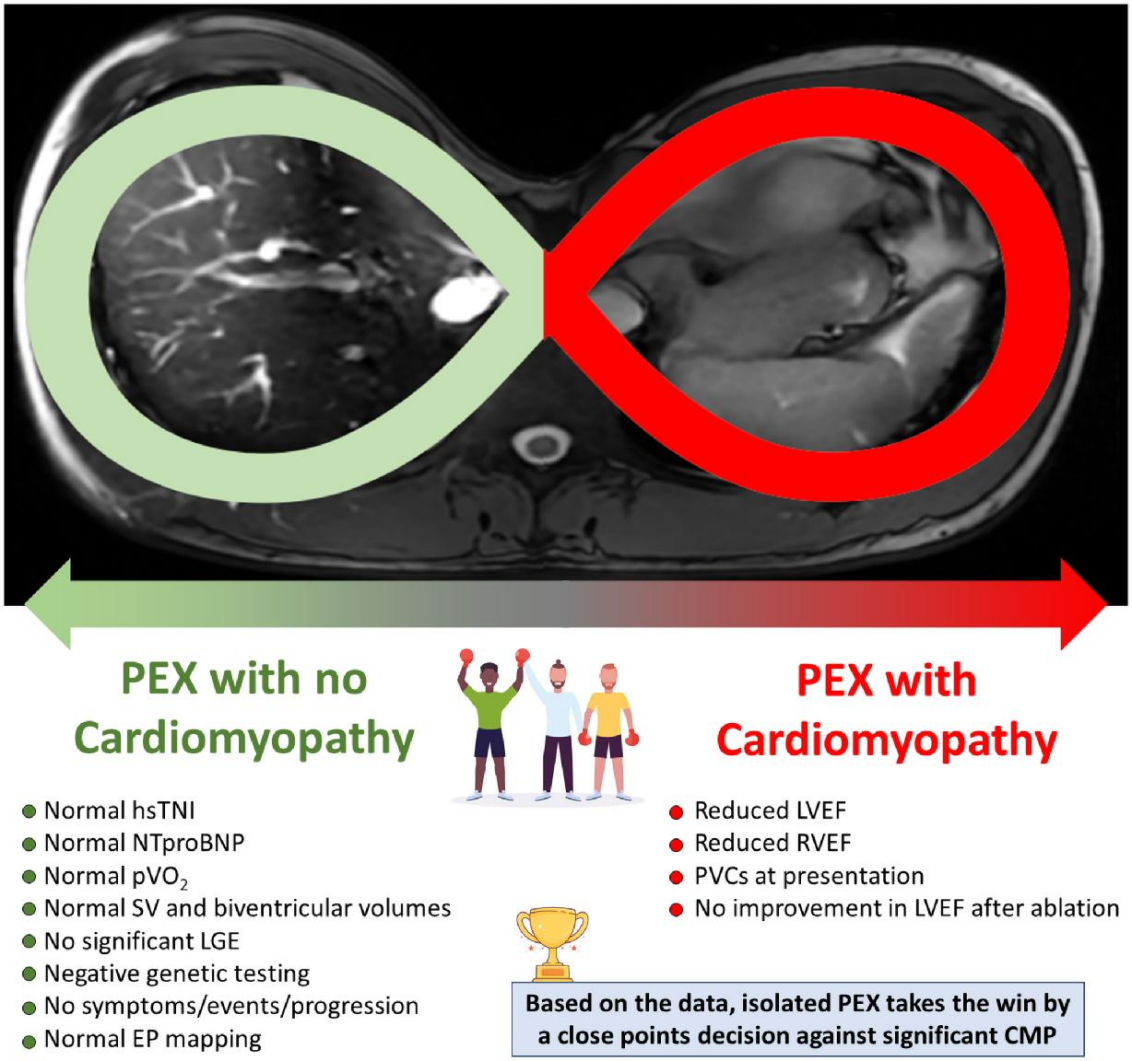
Trying to escape the PEX/CMP loop. The figure summarizes the main findings of this case and highlights how challenging the diagnosis of CMP can be in this context, despite the comprehensive use of multimodality imaging along with clinical, biomarker, and functional assessments. Moreover, it underscores the complexity involved in establishing a definitive diagnosis of ‘absence of CMP’, especially when such a decision has significant implications for recommendations regarding competitive sports participation. *Abbreviations*: PEX, Pectus Excavatum; CMP, Cardiomyopathy; hsTNI, High Sensitivity Troponin I; NTproBNP, N-terminal pro-B-type natriuretic peptide; pVO_2_, Peak Oxygen Uptake; SV, Stroke Volume; LGE, Late Gadolinium Enhancement; EP, Electrophysiology; LVEF, Left Ventricle Ejection Fraction; RVEF, Right Ventricle Ejection Fraction.

## Case description

**Figure 1:**
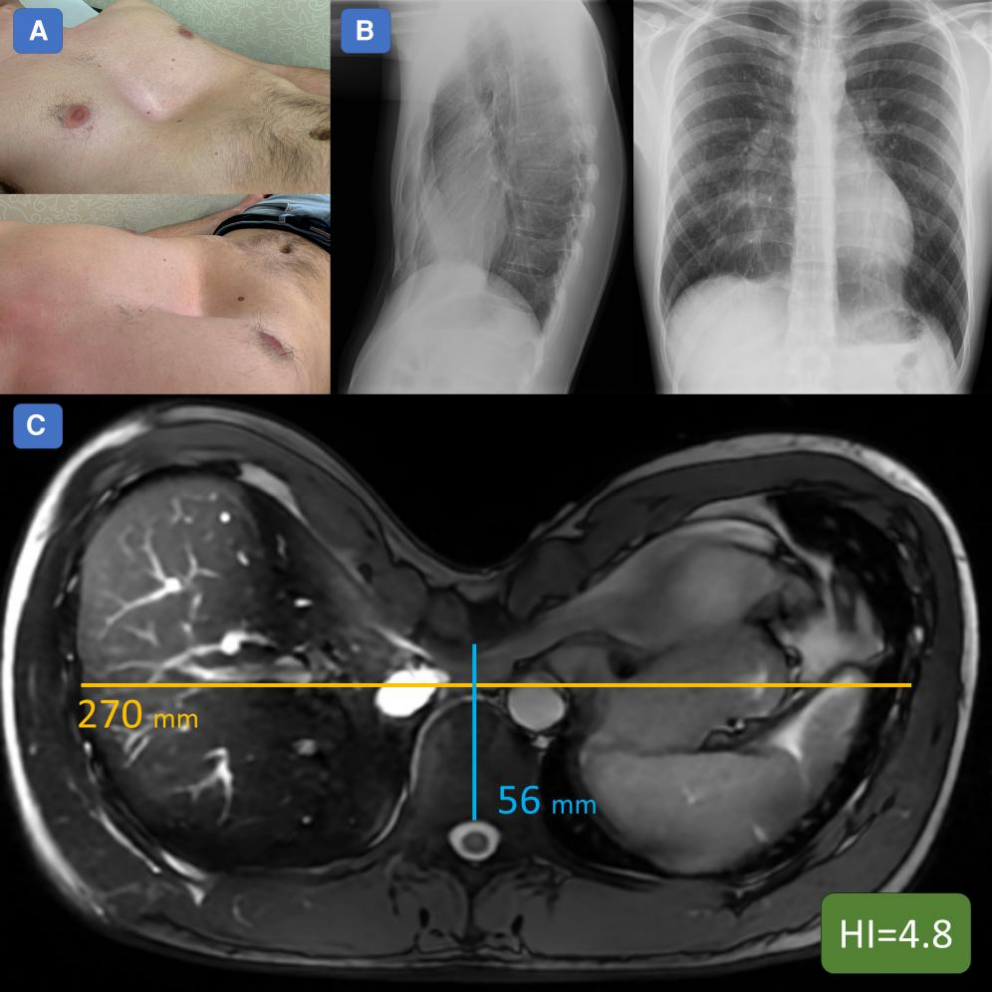
PEX anatomical and radiological findings. Panel A: PEX is clearly visible on physical examination. Panel B: Chest X-ray already shows typical features of PEX, including leftward displacement of the heart, blurring of the right cardiac border, and altered rib orientation; Panel C: CMR was also used to calculate the HI, which in this case was compatible with a severe form of PEX (4.8). *Abbreviations*: PEX, Pectus Excavatum; CMR, Cardiovascular Magnetic Resonance; HI, Haller Index.

**Figure 2:**
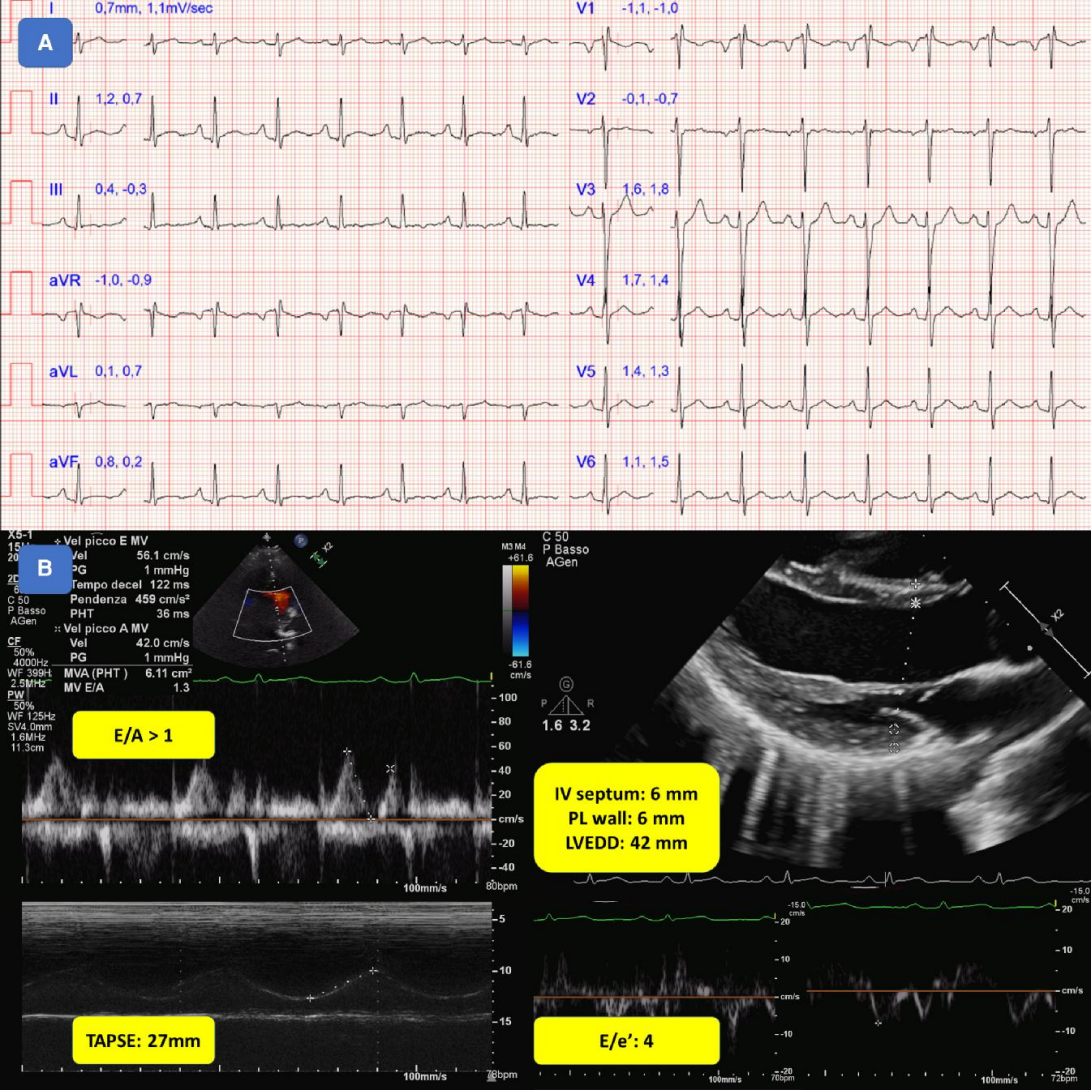
ECG and echocardiographic findings. Panel A: The ECG shows sinus rhythm with P pulmonale, normal AV conduction, mild RBBB, and a normal cardiac axis; Panel B: Despite suboptimal acoustic windows due to PEX, the echocardiogram clearly demonstrates normal filling pressures, normal TAPSE, and normal LV size and wall thickness. *Abbreviations*: RBBB, Right Bundle Branch Block; TAPSE, Tricuspid Annular Plane Systolic Excursion; PEX, Pectus Excavatum; IV, Interventricular; PL, Postero-Lateral; LVEDD: Left Ventricle End-Diastolic Diameter.

**Figure 3:**
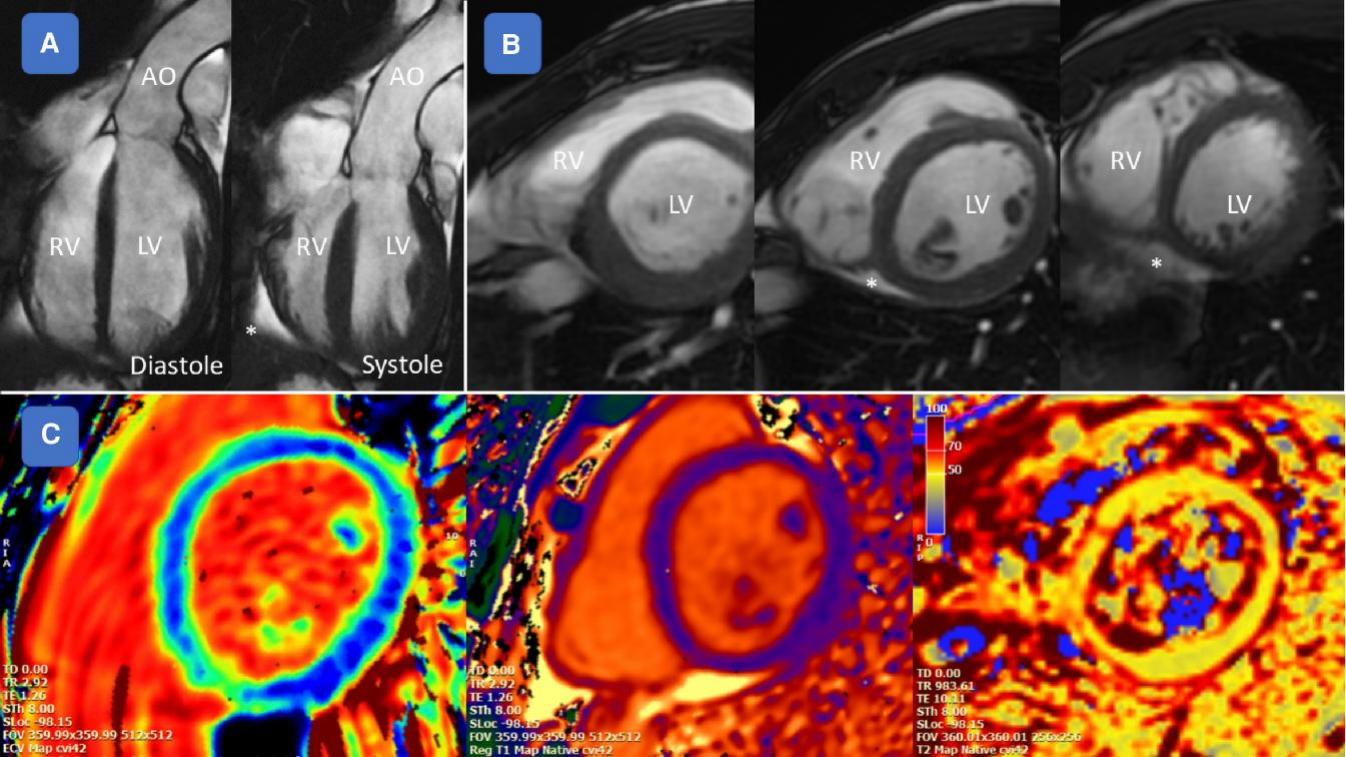
CMR findings. The patient underwent CMR, which showed normal LV volumes, slightly reduced biventricular systolic function (LVEF 48%; RVEF 43%), a small non-significant area of fibrosis at the inferior septal insertion between the interventricular septum and the RV, and mild diffuse PE. Panel A: Systolic and diastolic function assessment and evidence of mild PE; Panel B: Coronal views showing anatomical distortion, particularly of the RV, related to PEX; Panel C: Tissue characterization demonstrating normal ECV, T1, and T2 values in the investigated sequences. *Abbreviations*: CMR, Cardiac Magnetic Resonance; LV, left ventricle; RV, right ventricle; LVEF, Left Ventricle Ejection Fraction; RVEF, Right Ventricle Ejection Fraction AO, aorta; PE, pericardial effusion; ECV, extracellular volume; PEX, Pectus Excavatum.

**Figure 4:**
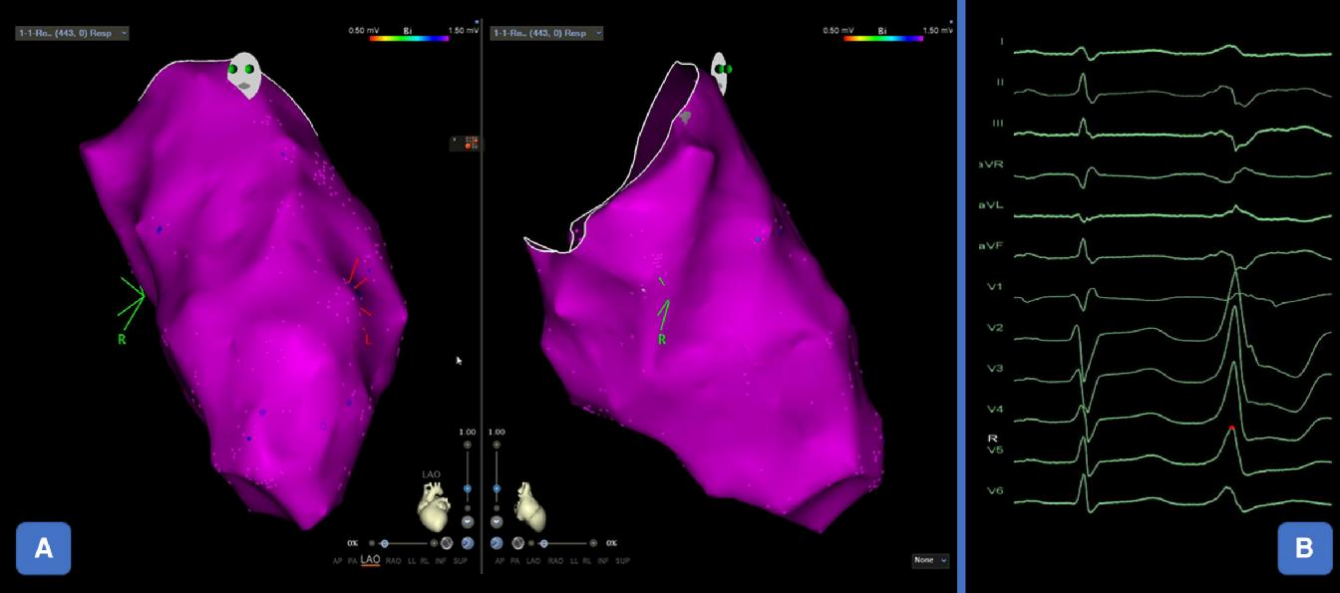
Electrophysiological study and LV mapping. Panel A: Electroanatomical mapping demonstrated normal endocavitary potentials, with no areas of abnormal substrate. Panel B: Electrophysiological study confirmed a monomorphic ventricular ectopic focus, which was successfully targeted by transcatheter ablation. *Abbreviations*: LV, Left Ventricle.

In March 2020, a fully asymptomatic 24-year-old patient, with a family history of sudden cardiac death (SCD) in a distant relative—but not in first-degree relatives—underwent competitive sports screening for soccer, which revealed frequent monomorphic PVCs with a right bundle branch block (RBBB) morphology and an intermediate axis. The physical examination was entirely normal except for difficulty in hearing heart sounds and the presence of a deep pectus excavatum (Haller index 4.8, *Figure 1*). The electrocardiogram (ECG) demonstrated sinus rhythm (SR) with non-specific repolarization abnormalities in the inferior leads and incomplete RBBB, while the echocardiogram, although limited due to a poor acoustic window related to chest configuration, showed normal RV longitudinal function, normal left ventricular (LV) diastolic function, and a mild reduction in left ventricle ejection fraction (LVEF) (*Figure 2*). A 24-hour ECG Holter Monitor recorded SR with 10 400 monomorphic isolated PVCs (corresponding to 13% of the total heart beats), with rare couplets (28 in 24 h). An exercise stress test showed disappearance of PVCs during maximal exercise. The patient underwent a CMR (August 2020) reporting normal LV volumes and slightly reduced biventricular systolic function (LVEF 48%; RVEF 43%), a small non-significant area of fibrosis in the inferior septal insertion between the interventricular septum and RV, and mild diffuse pericardial effusion (*Figure 3*). Given these data, a temporary cessation of sports activity was recommended. A further assessment, after 6 months, included a new CMR (April 2021) showing a slightly enlarged LV with septal dyskinesia and mildly reduced LVEF (46%), and a normal RV size with mild systolic dysfunction (RVEF 45%). The presence of PVCs was confirmed in another ECG Holter (May 2021) with the same predominant RBBB morphology (9736 isolated PVCs). An exercise stress test (May 2021) reported one PVC at peak exercise and a couplet during the recovery phase. Given the findings of frequent monomorphic and minimally repetitive PVCs, the patient, who was a young competitive athlete with no symptoms (he denied experiencing angina, dyspnoea, or syncopal episodes), in April 2022, successfully underwent transcatheter PVC ablation via an endocardial approach. Acute success was achieved without complications. Electrophysiological study showed no inducible ventricular arrhythmias, and LV mapping was negative for low-voltage areas or late/pathologic potentials (*Figure 4*).

In 2023, the patient, asymptomatic and in stable condition, underwent a follow-up echocardiogram showing normal LV dimensions with LVEF 46%; a 24-hour ECG Holter showed an almost complete disappearance of PVCs (4 PVCs in 24 h). A follow-up CMR (March 2023) indicated biventricular hypokinetic cardiomyopathy with stable reduction in LV and RV EF.

**Figure 5:**
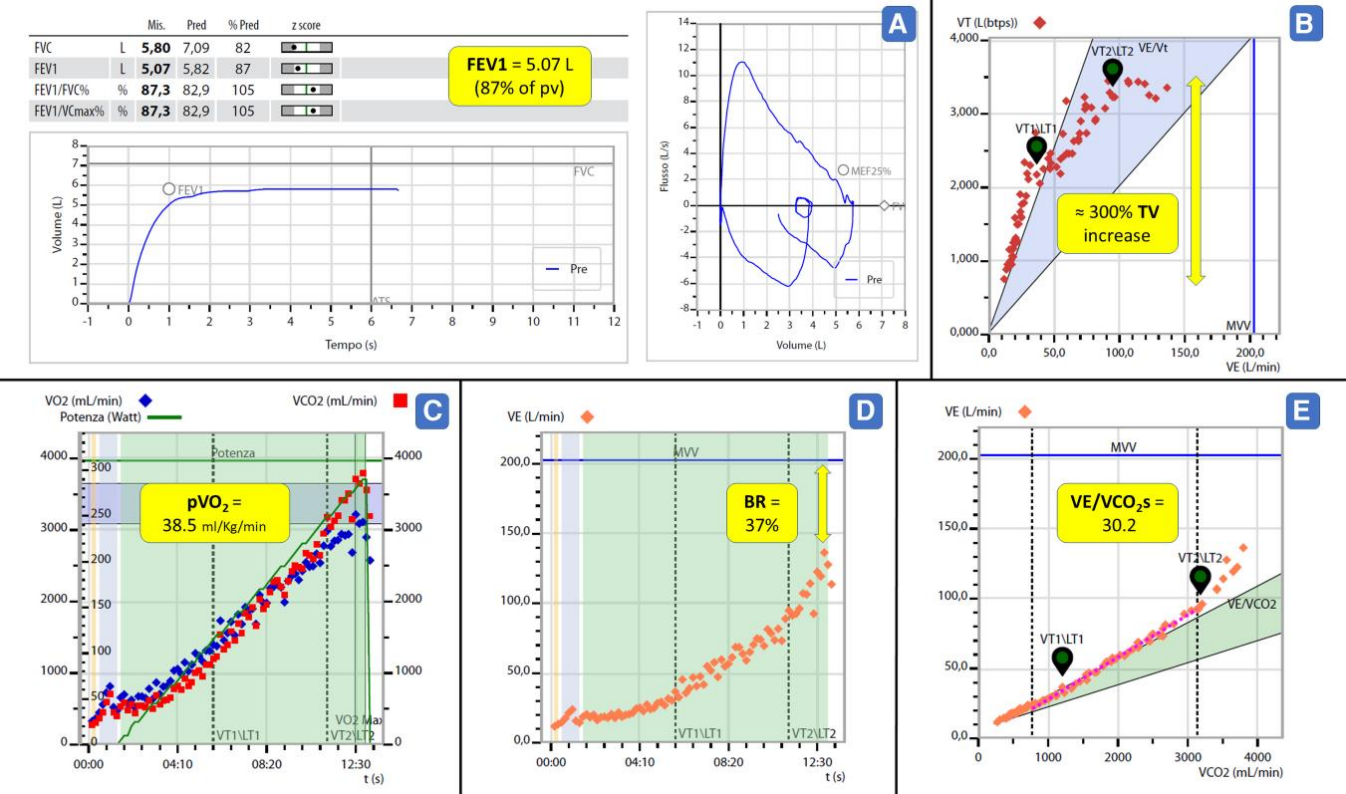
PFT and CPET findings. Panel A: PFT data showing FEV1 and FVC values within normal limits despite the presence of PEX, which can be associated with restrictive lung patterns; Panel B: Normal TV increase during exercise (∼300%), indicating preserved ventilatory capacity; Panel C: The patient achieved excellent pVO_2_ of 38.5 mL/kg/min, within normal range; Panel D: A wide respiratory reserve at the end of exercise is evident, excluding a ventilatory limitation to the exercise also at high intensity, with no arterial oxygen desaturation. Panel E: Normal ventilatory efficiency demonstrated by normal VE/VCO_2_ slope values. *Abbreviations*: CPET, Cardiopulmonary Exercise Testing; PFT, Pulmonary Function tTst; FEV1, Forced Expiratory Volume in 1 s; FVC, Forced Vital Capacity; TV, Tidal Volume; pVO_2_, Peak Oxygen Uptake; BR, Breathing Reserve; x\VE/VCO_2_s, Ventilatory Equivalent for Carbon Dioxide slope; PEX, Pectus Excavatum.

Blood tests (January 2024) were unremarkable—particularly normal blood counts, haemoglobin, creatinine, serum electrolytes, transaminase, TSH, NT-proBNP (< 35 ng/L), and high-sensitive Tropinine-I. The patient also underwent cardiopulmonary exercise testing (CPET) (December 2023), showing a maximal test with a normal peak VO_2_ of 38.5 mL/kg/min (84% of predicted according to the Hansen/Wasserman equation, 96% of the predicted according to the Neder equation, and VE/VCO_2_ at 30.2) (*Figure 5*). Few, rare, isolated monomorphic PVCs with RBBB morphology were noted during exercise. During the whole follow-up (2020–2024), the patient was not on any medications. In the meantime, the results of the genetic test became available, showing negative findings for pathogenic or likely pathogenic mutations.

The patient presented for cardiological consultation in March 2024, at the age of 24, in the context of the return to play (soccer) screening, following the sports physician’s advice.

## Discussion

We reported the case of an asymptomatic 24-year-old male with PEX and isolated mild LV dysfunction, persisting after frequent PVCs ablation, with no fibrosis at CRM, genetic mutations or familial predisposition, normal serum biomarkers, cardiac chambers volumes, functional capacity, and no pathological findings on ventricular myocardial mapping.

Given the clinical and follow-up data presented, the diagnosis of cardiomyopathy in this patient remains uncertain and warrants a careful evaluation of both supporting and opposing factors. While a mildly reduced LVEF has been observed, a closer examination of additional findings and their clinical relevance suggests that the criteria for a definitive diagnosis of cardiomyopathy may not be met.

Although CMR excluded a structural origin of PVCs, previous studies have shown that an increasing PVC burden, even when idiopathic and considered benign, can impair LV function.^4–6^ Due to the high suspicion of PVC-induced tachicardiomyopathy, the patient underwent cardiac ablation, as cardiac function usually recovers following PVC burden reduction.^7,8^ However, despite PVCs resolution, LVEF did not improve after 1 year, thus reducing the likelihood of a PVCs-induced cardiomyopathy.

The presence of mild associated abnormalities like the RBBB and minimal pericardial effusion, despite being observed also in different cardiac diseases, can be entirely explained by their high prevalence in patients with severe PEX^9^ and, in this clinical context, are not necessarily indicators of underlying cardiomyopathy.^10^ Indeed, Conte *et al*.^11^ recently described a 37% prevalence of pericardial effusion in unselected PEX subjects, not leading to significant clinical events. A similar prevalence of pericardial effusion was reported in a small trial by Oezcan *et al.,*^12^ together with a 50% prevalence of incomplete RBBB. Additionally, the absence of a progressive LV dilation in our patient over time is reassuring, as cardiomyopathy often presents with ventricular enlargement. Even considering their low negative predictive value,^13^ genetic testing also supports a benign interpretation, as no pathogenic or likely pathogenic mutations were identified. This finding is crucial in a young patient with a possible family history of SCD in a distant relative, as genetic cardiomyopathies frequently present with identifiable mutations.^14^ The totally silent clinical condition, together with the absence of family history of SCD in first-degree relatives and of genetic mutation, reduces the likelihood of a hereditary cardiomyopathic condition and, more importantly, speaks in favour of a good prognosis.

Moreover, the presence of true myocardial structural alterations is more than questionable, given a normal electroanatomical mapping during the invasive electrophysiology study and the absence of significant fibrosis at CMR (often observed in cardiomyopathy and a hallmark of myocardial disease). On top, blood markers, including troponin and NT-proBNP, were consistently within normal ranges, indicating an absence of ongoing myocardial injury or increased cardiac wall stress. The patient's preserved functional capacity on CPET supports a functional reserve inconsistent with cardiomyopathy, especially without medical therapy.

It is important to note that the LVEF measurement alone, while slightly reduced, may not provide a reliable indication of this patient's true stroke volume (SV) or functional capacity.^15^ This could be particularly true and relevant in patients with peculiar anatomical conditions (i.e. PEX), when EF measurements can be challenging to interpret due to geometrical variations (or due to poor acoustic window in the case of echocardiography), and EF may poorly correlate with actual SV or physical performance.

On the other side, the PEX, *per se,* has been related to different degrees of cardiac function impairment. Indeed, Zens *et al*. demonstrated a significant LV and RV impairment in a cohort of 345 young PEX patients who underwent CMR.^2^ Specifically, an abnormal RV and LV function (defined as a reduction in EF) was present in 32% and 18% of the cases, respectively. Moreover, among these patients, an inverse correlation between Haller index and RVEF or LVEF had been demonstrated. These data were in line with previous smaller trial.^12^ Although surgical correction of chest defect with the aim of improving cardiac function is debated,^15^ some studies^16,17^ underlined that LV functional alterations promptly reverted after surgery, supporting the hypothesis of ‘anatomical dysfunction,’ as patients with PEX showed significant alterations of cardiac morphology and function that manifest as an exaggerated interventricular dependence.^18^ Moreover, the rise in negative thoracic pressure could play a role after surgery. In 20 adult patients, Neviere *et al*. analysed inspiratory muscle strength before and 1 year after chest surgery, demonstrating a significant increase along with an exercise of O_2_-pulse and peakVO_2_, suggesting an improvement in the capacity of the inspiratory muscles to produce an intrathoracic negative pressure, thus increasing cardiac filling and improving biventricular stroke.

Our finding should also be commented with respect to the recent ESC guidelines on cardiomyopathies,^13^ which identified a new phenotype (i.e, non-dilated left ventricular cardiomyopathy, NDLVC) that is defined as the presence of non-ischaemic LV scarring or fatty replacement regardless of the presence of global or regional wall motion abnormalities, or isolated global LV hypokinesia without scarring. This definition, while important for identifying cases of cardiomyopathy that were previously ‘orphaned’ due to a lack of appropriate phenotypic characterization, is nonetheless rather broad and general. Under the NDLVC umbrella, cardiomyopathies that differ significantly in terms of epidemiology and clinical-therapeutic approach can be grouped together.^19^ Furthermore, certain specific cases, such as that of our patient, risk being equated with much more severe cardiac conditions, with the result of impacting clinical management and quality of life, as though a conditioning grant for participation in competitive sports activities.

### Isolated systolic dysfunction and competitive sports. Return to play?

#### The Italian perspective

A nationwide preparticipation screening program (PPS) was introduced in Italy in 1982, resulting in an 89% decrease in the incidence rate of SCD among young competitive athletes aged 12–35 years, as demonstrated in the Veneto region.^3^ However, the 2023 edition of Italian Cardiological Guidelines (COCIS) for Competitive Sport Eligibility^20^ was published prior to the 2023 ESC Guidelines on the management of cardiomyopathies and therefore does not include NDLVC. Consequently, this patient would be classified and managed according to the recommendations for DCM.

Competitive sports activities are not recommended (class of recommendation III C) in patients with DCM and one or more of the risk markers of SCD or haemodynamic deterioration: symptoms, particularly syncope; family history of SCD before age 40 in first-degree relatives, Atrial Fibrillation (AF), paroxysmal supraventricular tachycardia, frequent and polymorphic PVCs, non-sustained ventricular tachycardia (≥3 beats, >120 bpm); moderate-to-severe LV dysfunction (LVEF <45%), segmental LV akinesia or dyskinesia, insufficient improvement in LVEF (<15%), or an abnormal blood pressure response (less than a 20 mmHg increase at peak exercise) during exercise stress testing, extensive areas of LGE on CMR, pathogenic or likely pathogenic mutations in genes associated with a high risk of adverse outcomes (such as LMNA, SCN5A, PLN, TMEM43, FLNC, RBM20, and DSP). Asymptomatic patients with a diagnosis of DCM with no risk markers may engage in competitive sports activities after an accurate evaluation in experienced centres, considering the discipline that the patient wishes to practice (class of recommendation II C). For these reasons, the patient of our case was considered ineligible for competitive sports participation.

#### The United Kingdom perspective

In the United Kingdom (UK), eligibility for competitive sports in athletes with potential cardiac abnormalities is addressed through a nuanced, risk-based approach rather than rigid exclusion criteria. Clinical decision-making is guided by international recommendations,^21^ specialists' expertise, and a personalized, patient-centred model, with a strong emphasis on shared decision-making between clinicians and athletes. This approach allows for individualized assessment and management, aiming to ensure both athlete autonomy and safety. The 2022 UK Cardiovascular Consensus Statement on risk stratification in sport^22^ emphasizes shared decision-making, considering not only structural findings but also the athlete’s symptoms, functional capacity, and personal values. In this case, the athlete has a mildly reduced LV ejection fraction (∼46%), which is not typical for physiological remodelling, particularly given the volume of training he is undergoing. However, is entirely asymptomatic, the EF remained stable, there were no inducible arrhythmias or structural myocardial scarring, normal cardiopulmonary performance, and no high-risk genetic findings. In the absence of clear indicators of progressive disease or arrhythmic vulnerability, a multidisciplinary evaluation—including a sports cardiologist, an electrophysiologist, and a sports physician—might support a return to competitive football, especially given the successful suppression of PVCs post-ablation.

The UK model would also consider the psychological and social impact of sport disqualification. After full risk disclosure, if the athlete expresses a strong wish to return, participation may be granted under regular follow-up, including annual ECG Holter, CMR every 1–2 years, CPET, and stress testing tailored to the athlete’s sport. This personalized, collaborative approach contrasts with more prescriptive models and reflects a broader shift in sports cardiology toward empowering the athlete in decision-making.

#### The Dutch perspective

The Netherlands adopts a similarly flexible and patient-centred approach in evaluating eligibility for sports participation in athletes with cardiac anomalies. Dutch guidelines, developed through collaboration between the Nederlandse Vereniging voor Cardiologie (NVVC) and sports medicine societies, emphasize functional status over static structural metrics, in line with ESC recommendations but interpreted through a pragmatic lens.^23^ Key principles in the Dutch approach include risk stratification based on comprehensive evaluation, not isolated parameters such as LVEF alone. Emphasis on functional capacity (e.g. CPET), arrhythmia burden, and symptomatology. Consideration of the athlete’s own preferences and level of sport (recreational vs. elite competitive). In this patient’s case—mild, stable LV dysfunction, no residual arrhythmias, no genetic markers or myocardial fibrosis, and normal performance on CPET—the Dutch approach would likely view the profile as low arrhythmic risk, particularly in the absence of red flags such as syncope, VT, or progressive myocardial changes. Athletic clearance might be granted with informed consent, ongoing structured follow-up, and sport-specific evaluation, particularly considering football’s intermittent high-exertion demands. The Dutch system supports ‘conditional eligibility,’ meaning that athletes can be cleared for participation with agreed-upon safeguards and monitoring protocols, reinforcing the idea that risk management does not equal risk elimination, but rather informed engagement in physical activity.

*Table 1* summarized the slight differences in approaching the same condition in the three different countries considered.

**Table 1:** Comparative international approaches to eligibility for competitive sports in suspected NDLVC with mild LV dysfunction

Domain	IT Italy	GB United Kingdom	NL The Netherlands	US The USA
Clinical approach	Guideline-driven (COCIS); structured and restrictive.	Holistic and risk-adaptive; emphasizes shared decision-making.	Function-oriented, athlete-centred; multidisciplinary risk stratification.	Patient-centred, strong focus on shared decision-making
Thresholds for LVEF	<45% with risk factors = exclusion (Class III C); 45%–50% = discretionary.	No rigid cutoffs: mild dysfunction considered in context of symptoms and arrhythmic profile.	LVEF is not used in isolation; contextualized with broader clinical data.	No set cutoffs, LVEF is considered in the context of additional risk markers.
Ventricular ectopy	Frequent PVCs with LV dysfunction = high-risk marker.	Suppressed PVCs post-ablation = low-risk if asymptomatic and well-functioning.	Isolated PVCs are often tolerated; focus on burden, morphology, and inducibility.	Absence of PVCs post ablation, both at rest and during exercise = low risk marker
Imaging and EP study	LGE and electroanatomic mapping abnormalities = disqualifying.	Normal CMR/EP mapping = reassuring; permits eligibility in selected cases.	Absence of fibrosis/pathologic EP findings = strong argument for clearance.	Normal CMR/EP study = low risk marker
CPET and functional markers	Considered ancillary; low weight vs. structural markers.	Central to prognosis; good capacity suggests favourable outcome.	Primary tool; high performance correlates with eligibility.	Not specifically considered in the recommendations, but generally a normal capacity relates to favourable outcome.
Athlete’s role in decision	Limited; eligibility is dictated by guidelines and physician assessment.	Active participant; decision shared after structured counselling.	Integral to process; autonomy emphasized alongside medical expertise.	Central to the American recommendations following specialist counselling
Expected decision in the presented case	Disqualified from competitive sports due to LVEF <50% and suspicion of cardiomyopathy.	Conditionally eligible in absence of red flags, with surveillance in a reference centre.	Likely eligible, pending functional performance and regular follow-up within a multidisciplinary team.	Likely eligible, pending regular follow-up in specialized centers

Abbreviations: CPET, cardiopulmonary exercise test; EP, electrophysiology; LGE, late gadolinium enhancement; LVEF, left ventricular ejection fraction; NDLVC, non-dilated left ventricular cardiomyopathy; PVC, premature ventricular contraction.

### The USA prospective

The latest statement from the American College of Cardiology regarding competitive sport participation^24^ emphasizes the critical role of shared decision-making. Rather than enforcing rigid exclusion criteria, the document serves as a framework to guide clinicians in evaluating the athlete’s risk related to sport participation. In this case, the reduction in EF is inconsistent with what would be expected from training induced physiological remodelling, especially because no reverse remodelling has been observed after the extensive detraining. However, the reduction may be, at least partially, explained by his PEX. Furthermore, the EF has remained stable during follow-up, he is asymptomatic, his arrhythmic burden is negligible after the ablation, and no inducible arrythmias have been observed during exercise testing. The CMR failed to show any myocardial scaring, and the CPET demonstrated a normal functional capacity. In addition, the athlete does not harbour any high-risk genetic mutation. Although cardiomyopathy cannot be definitively ruled out, the overall risk of SCD is likely to be perceived as low; thus, he may be deemed eligible for competitive sport participation following a comprehensive assessment at a specialized centre and extended discussion of the potential risks. Ongoing surveillance would remain essential to monitor for any progression of left ventricular dysfunction.

## Conclusions

This case highlights the diagnostic and management challenges in athletes with mildly reduced systolic function and severe pectus excavatum, where anatomical distortion can mimic cardiomyopathy. Despite a thorough evaluation, the diagnosis remained uncertain, raising concerns about rigid interpretations of functional impairment in the absence of clear structural or genetic findings. Differences in sports eligibility policies across countries reveal a need for more harmonized and nuanced approaches, especially in borderline cases. Exploring shared decision-making models—supported by expert centres and structured follow-up—may offer a balanced alternative, reducing the physical and psychological burden of exclusion from competitive team sports. The evolving definition of NDLVC is clinically relevant, but care must be taken not to fall into diagnostic loops that hinder effective, individualized strategies.

## Data Availability

The data underlying this article are not publicly available due to patient privacy and ethical restrictions but are available from the corresponding author upon reasonable request.

## Lead author biography

Massimo Mapelli, MD, PhD, is a cardiologist and Researcher at the Heart Failure Unit of Centro Cardiologico Monzino, IRCCS, and at the University of Milan, Italy. His clinical and research activities focus on heart failure, cardiopulmonary exercise testing, and exercise physiology, with particular interest in the integration of hemodynamic and metabolic responses during exercise. Dr. Mapelli has authored more than 200 peer-reviewed publications and has contributed to several national and international research projects. He is actively involved in mentoring young investigators and promoting translational research bridging clinical cardiology and exercise science.

## Notes

**Consent:** A patient informed consent was obtained to publish this case report.

## References

[1] Williams AM, Crabbe DCG. Pectus deformities of the anterior chest wall. Paediatr Respir Rev 2003;4:237–242. 12880759 10.1016/s1526-0542(03)00053-8

[2] Saleh RS, Finn JP, Fenchel M, Moghadam AN, Krishnam M, Abrazado M, et al Cardiovascular magnetic resonance in patients with pectus excavatum compared with normal controls. J Cardiovasc Magn Reson 2010;12:73. 21144053 10.1186/1532-429X-12-73 PMC3022801

[3] Corrado D, Basso C, Pavei A, Michieli P, Schiavon M, Thiene G. Trends in sudden cardiovascular death in young competitive athletes after implementation of a preparticipation screening program. JAMA 2006;296:1593–1601. 17018804 10.1001/jama.296.13.1593

[4] Chugh SS, Shen WK, Luria DM, Smith HC. First evidence of premature ventricular complex-induced cardiomyopathy: a potentially reversible cause of heart failure. J Cardiovasc Electrophysiol 2000;11:328–329. 10749356 10.1111/j.1540-8167.2000.tb01802.x

[5] Agarwal V, Vittinghoff E, Whitman IR, Dewland TA, Dukes JW, Marcus GM. Relation between ventricular premature complexes and incident heart failure. Am J Cardiol 2017;119:1238–1242. 28214002 10.1016/j.amjcard.2016.12.029

[6] Niwano S, Wakisaka Y, Niwano H, Fukaya H, Kurokawa S, Kiryu M, et al Prognostic significance of frequent premature ventricular contractions originating from the ventricular outflow tract in patients with normal left ventricular function. Heart 2009;95:1230–1237. 19429571 10.1136/hrt.2008.159558

[7] Latchamsetty R, Yokokawa M, Morady F, Kim HM, Mathew S, Tilz R, et al Multicenter outcomes for catheter ablation of idiopathic premature ventricular complexes. JACC Clin Electrophysiol 2015;1:116–123. 29759353 10.1016/j.jacep.2015.04.005

[8] Ling Z, Liu Z, Su L, Zipunnikov V, Wu J, Du H, et al Radiofrequency ablation versus antiarrhythmic medication for treatment of ventricular premature beats from the right ventricular outflow tract: prospective randomized study. Circ Arrhythm Electrophysiol 2014;7:237–243. 24523413 10.1161/CIRCEP.113.000805

[9] Farina JM, Yinadsawaphan T, Jaroszewski DE, Aly MR, Botros M, Cheema KP, et al The electrocardiographic manifestations of pectus excavatum before and after surgical correction. J Electrocardiol 2024;82:19–26. 38000149 10.1016/j.jelectrocard.2023.11.007

[10] Lazaros G, Lazarou E, Tsioufis P, Soulaidopoulos S, Iliakis P, Vlachopoulos C, et al Chronic pericardial effusion: current concepts and emerging trends. Expert Rev Cardiovasc Ther 2022;20:363–376. 35524164 10.1080/14779072.2022.2075346

[11] Conte E, Agalbato C, Lauri G, Mushtaq S, Carollo C, Bonomi A, et al Prevalence and prognosis of pericardial effusion in patients affected by pectus excavatum: a case-control study. Int J Cardiol 2021;344:179–183. 34626741 10.1016/j.ijcard.2021.10.005

[12] Oezcan S, Attenhofer Jost CH, Pfyffer M, Kellenberger C, Jenni R, Binggeli C, et al Pectus excavatum: echocardiography and cardiac MRI reveal frequent pericardial effusion and right-sided heart anomalies. Eur Heart J Cardiovasc Imaging 2012;13:673–679. 22298154 10.1093/ehjci/jer284

[13] Arbelo E, Protonotarios A, Gimeno JR, Arbustini E, Barriales-Villa R, Basso C, et al 2023 ESC guidelines for the management of cardiomyopathies. Eur Heart J 2023;44:3503–3626. 37622657 10.1093/eurheartj/ehad194

[14] Paldino A, De Angelis G, Merlo M, Gigli M, Dal Ferro M, Severini GM, et al Genetics of dilated cardiomyopathy: clinical implications. Curr Cardiol Rep 2018;20:83. 30105555 10.1007/s11886-018-1030-7

[15] Maagaard M, Heiberg J. Improved cardiac function and exercise capacity following correction of pectus excavatum: a review of current literature. Ann Cardiothorac Surg 2016;5:485–492. 27747182 10.21037/acs.2016.09.03 PMC5056930

[16] Sonaglioni A, Nicolosi GL, Trevisan R, Lombardo M, Grasso E, Gensini GF, et al The influence of pectus excavatum on cardiac kinetics and function in otherwise healthy individuals: a systematic review. Int J Cardiol 2023;381:135–144. 37003372 10.1016/j.ijcard.2023.03.058

[17] Krueger T, Chassot PG, Christodoulou M, Cheng C, Ris HB, Magnusson L. Cardiac function assessed by transesophageal echocardiography during pectus excavatum repair. Ann Thorac Surg 2010;89:240–243. 20103244 10.1016/j.athoracsur.2009.06.126

[18] Deviggiano A, Vallejos J, Vina N, Martinez-Ferro M, Bellia-Munzon G, Carrascosa P, et al Exaggerated interventricular dependence among patients with pectus Excavatum: combined assessment with cardiac MRI and chest CT. AJR Am J Roentgenol 2017;208:854–861. 28140622 10.2214/AJR.16.17296

[19] Monda E, Murredda A, Rubino M, Diana G, Palmiero G, Verrillo F, et al Aetiology and clinical manifestations of patients with non-dilated left ventricular cardiomyopathy. Eur J Heart Fail 2024;26:2579–2581. 39300765 10.1002/ejhf.3470

[20] Zeppilli P, Biffi A, Cammarano M, Castelletti S, Cavarretta E, Cecchi F, et al Italian cardiological guidelines (COCIS) for competitive sport eligibility in athletes with heart disease: update 2024. Minerva Med 2024;115:533–564. 39435618 10.23736/S0026-4806.24.09519-3

[21] Pelliccia A, Sharma S, Gati S, Bäck M, Börjesson M, Caselli S, et al 2020 ESC guidelines on sports cardiology and exercise in patients with cardiovascular disease. Eur Heart J 2021;42:17–96.Erratum in: Eur Heart J. 2021 Feb 1;42(5):548–549. doi: 10.1093/eurheartj/ehaa835. 32860412 10.1093/eurheartj/ehaa605

[22] Reid H, Ridout AJ, Tomaz SA, Kelly P, Jones N. Physical activity risk consensus group. Benefits outweigh the risks: a consensus statement on the risks of physical activity for people living with long-term conditions. Br J Sports Med 2022;56:427–438. 34649919 10.1136/bjsports-2021-104281 PMC8995821

[23] van Hattum JC, Verwijs SM, Rienks R, Bijsterveld NR, de Vries ST, Pinto YM, et al The Netherlands sports cardiology map: a step towards sports cardiology network medicine for patient and athlete care. Neth Heart J 2021;29:129–134. 33355906 10.1007/s12471-020-01530-x PMC7904973

[24] Kim JH, Baggish AL, Levine BD, Ackerman MJ, Day SM, Dineen EH, et al Clinical considerations for competitive sports participation for athletes with cardiovascular abnormalities: a scientific statement from the American Heart Association and American College of Cardiology. J Am Coll Cardiol 2025;85:1059–1108. 39976316 10.1016/j.jacc.2024.12.025 PMC12145891

